# Ethylene-Induced Vinblastine Accumulation Is Related to Activated Expression of Downstream TIA Pathway Genes in *Catharanthus roseus*

**DOI:** 10.1155/2016/3708187

**Published:** 2016-05-29

**Authors:** Xi Wang, Ya-Jie Pan, Bo-Wen Chang, Yan-Bo Hu, Xiao-Rui Guo, Zhong-Hua Tang

**Affiliations:** ^1^Key Laboratory of Forest Plant Ecology, Northeast Forestry University, Harbin 150040, China; ^2^College of Life Science, Northeast Forestry University, Harbin 150040, China

## Abstract

We selected different concentrations of ethephon, to stress* C. roseus*. We used qRT-PCR and HPLC followed by PCA to obtain comprehensive profiling of the vinblastine biosynthesis in response to ethephon. Based on our findings, the results showed that the high concentration of ethephon had a positive effect at both transcriptional and metabolite level. Meanwhile, there was a remarkable decrease of hydrogen peroxide content and a promoted peroxidase activity in leaves. The loading plot combination with correlation analysis suggested that* CrPrx1* could be regarded as a positive regulator and interacts with ethylene response factor (*ERF*) to play a key role in vinblastine content and peroxidase (POD) activity. This study provides the foundation for a better understanding of the regulation and accumulation of vinblastine in response to ethephon.

## 1. Introduction 

**Figure 1:**
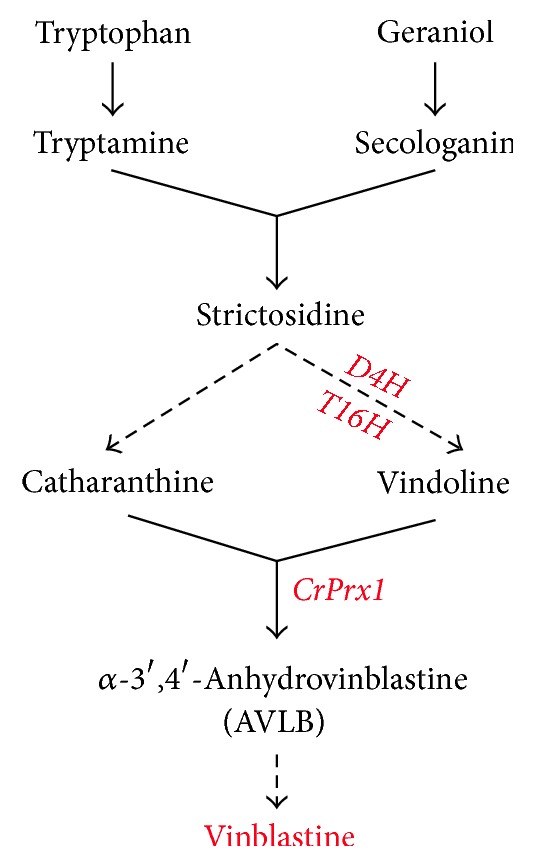
The pathway of terpenoid indole alkaloids biosynthesis in* Catharanthus roseus.*

*Catharanthus roseus* (L.) G. Don., also called Madagascar periwinkle, is widely used in studies as a model of medical plant for many kinds of terpenoid indole alkaloids (TIAs) [1]. Because of the antineoplastic activity in the treatment of many cancers, some of them have medicinal and scientific research value, such as vinblastine [2, 3]. The early stages of vinblastine biosynthesis in* C. roseus* involve the formation of tryptamine from tryptophan and its condensation with secologanin to produce the central intermediate strictosidine, the common precursor for the monoterpenoid indole alkaloids, vindoline and catharanthine (Figure 1) [3]. These two monomeric alkaloids will then be enzymatically condensed to form the bisindole alkaloid vinblastine (Figure 1) [4].

Various proteins are involved in plant defense and secondary metabolic responses. Among these proteins, class III plant peroxidases (EC 1.11.1.7) are well known. As is reported, there are various abbreviations used for class III plant peroxidases such as POD, POX, and PRX [5]. Peroxidases (POD), always known as a type of antioxidant, are also involved in the biosynthesis of secondary metabolites for catalyzing production of vinblastine. Although the functions of POD are not well understood in plants, the enzyme has been recognized with medicinal properties [5]. The above statement is the case of the TIAs of* Catharanthus roseus* [6]. Sottomayor et al. found that peroxidase was purified to homogeneity and a channeling mechanism was proposed for the peroxidase mediated-vacuolar synthesis of *α*-3′,4′-anhydrovinblastine (AVLB) [4, 6]. AVLB can be converted into vinblastine, which is biosynthesized through coupling of the monomeric precursors vindoline and catharanthine [7].

The POD encoded by* CrPrx1* gene is also a multifunctional enzyme that has another function of cleaning up the oxidation of small molecules including H_2_O_2_ in the cells. H_2_O_2_ is an electron-accepting substrate for a wide variety of peroxidase-dependent reactions; thus, POD is generally considered to be merely a ROS-detoxifying enzyme [8]. The breakdown of H_2_O_2_ accumulation by the POD reaction is highly active especially in the presence of ROS-scavenging POD substrates [9]. They have also been involved in secondary metabolism, in root elongation, and in hydrogen peroxide scavenging and production; furthermore, they are thought to play significant roles for plants in stress resistance and adaptation [6, 10–16]. Although the detailed correlation between* CrPrx1* transcript and alkaloid levels was not clear, evidence further supported an important role for POD and* CrPrx1* in indole alkaloid biosynthesis, revealing the potential of* CrPrx1* as a molecular tool for the manipulation of alkaloid metabolism. We have obtained several partial nucleotide sequences which were used to isolate the downstream TIA pathway genes (*DAT, D4H, T16H*, and* CrPrx1*) and an ethylene response factor gene (*ERF*) by a RT-PCR to figure out the relationship between vinblastine biosynthesis and the expression of genes under ethylene-regulated control.

Plant secondary metabolites play critical roles in plant-environment interactions. Thus, there are many environmental conditions that could frequently affect their synthesis, such as salinity, light, nitrate, and potassium [17–21]. Ethylene, known as a phytohormone, plays an important role in regulating plant growth and development among the whole life cycle of the plant [22–24]. Some research reported that the biotic or abiotic stress-induced expression of* CrPrx1* is conferred by the nature of the 5′ flanking regions of the genes that contain many kinds of potential stress-responsive* cis*-elements [25]. Physical injuries promoted an increase of production of nicotine which resulted from the transcriptional activation of the putrescine* N*-methyltransferase gene and this gene encodes a regulatory enzyme in nicotine biosynthesis [26, 27]. In this way, we mainly discuss the relationship between the accumulation of vinblastine and transcriptional level of* CrPrx1*, which plays a putative role in catalyzing the condensation of vindoline and catharanthine.

## 2. Materials and Methods

### 2.1. Plant Materials and Cultivation Methods

*C. roseus* seedlings were germinated in perlite with distilled water until having grown out two pairs of leaves and then transferred to standalone plates with Hoagland nutrient solution. The seedlings were kept in a growth chamber at 28°C under a 16 h photoperiod.

### 2.2. Ethephon Treatment

Seedlings from the three-month-old plants were harvested, and 3 fully expanded leaves of seedlings were randomly selected and subjected to hydroponic treatment. For ethephon treatment, seedlings were cultivated in the Hoagland nutrient solution containing ethephon. We illustrated all the concentrations of ethephon used in our experiment as 45 *μ*M, 60 *μ*M, and 100 *μ*M and the treatment lasted 4 days.

### 2.3. Ethylene Release Measurement

For measurement of ethylene released from grown plants, plants with or without ethephon treatment grew in a Bunsen beaker and the Bunsen beaker was sealed to air tightness with a plastic membrane. Ethylene that was released by treated plants was determined by gas chromatography (Agilent Technologies, 7890A GC Systems). One milliliter of air from each container was taken to detect the presence of ethylene.

### 2.4. Alkaloid Analysis

Dry leaf powder (0.3 g) was dissolved in 10 mL absolute methanol (analytical grade) for extraction of vinblastine, vindoline, and catharanthine. Low-frequency ultrasonication (250 W, 40 kHz) was used to extract the alkaloids for 20 min. The methanol extract was centrifuged at 8000 ×g for 10 min, concentrated to 1 mL, and analyzed by HPLC (Jasco, VG, England) equipped with a Waters ODS C_18_ reversed-phase column (250 × 4.6 mm, 5 *μ*m) and a photodiode-array detector at 220 nm. Sample injection volume was 10 *μ*L at a flow rate of 1.5 mL min^−1^. Samples were applied in triplicate for quantification of vinblastine. The alkaloids were quantified by using regression equation of calibration curve.

### 2.5. Analysis of Redox State

Endogenous H_2_O_2_ concentrations were determined according to Patterson et al. [28]. Hydrogen peroxide coupled with titanium sulfate generating superoxide-titanium which was a yellow precipitate. Superoxide-titanium was dissolved by sulfuric acid, and the color of the solution had a linear relation with hydrogen peroxide concentration. The absorbance was read at 415 nm using an ultraviolet-visible spectrophotometer (UV-2550, Shimadzu, Japan). H_2_O_2_ was determined from a calibration curve.

Accurately measured 0.5 g of* C. roseus* leaves for the determination of peroxidase activity (POD, EC 1.11.1.7) was homogenized to a fine powder under liquid nitrogen. Then, the enzyme was extracted from 5 mL PBS (pH 7.0) with 1.0 mM EDTA, 1.0 mM ascorbic acid, and 10 g/L PVP. The homogenate was centrifuged under −4°C and 10000 g/min for 30 min. A hundred-microliter sample solution was added to 1.8 mL hydrochloric acid buffer solution, 1.0 mL guaiacol, and 0.1 mL hydrogen peroxide. Peroxidase activities were assayed in UV-2550 (Shimadzu, Japan) at 460 nm for 3 min [29].

### 2.6. RNA Extraction and RT-PCR

Total RNA was extracted from 50–100 mg samples (both leaves and roots) by TRIzol reagent and quantified by a NanoDrop ND-1000 spectrophotometer (NanoDrop Technologies) with absorbance at 260 nm and ethidium bromide (EB) stained test agarose gel electrophoresis used to verify the quality. cDNA was synthesized from total RNA (2 *μ*g) using ReverTra Ace QPCR RT Kit (Toyobo, Japan) according to the manufacturer's instructions, using oligo(dT) as the primer. qRT-PCR analysis using cDNA as template and gene-specific primers was performed using a SYBR Premix Ex Taq (TaKaRa, Japan). Gene-specific primers used are listed in Table 1 (from ExPlant Technologies B.V.).

PCR was performed at 94°C for 5 min and then at 94°C for 30 s, 60°C for 30 s, and 72°C for 30 s, on 80°C reading plate for 1 s for 35 cycles, and remained at 72°C for 2 min. Reactions were repeated three times for each sample to ensure the reproducibility of the results.* RPS9* gene was used as an internal control. After PCR reaction, a melting curve was obtained by Opticon version 3. The comparative CT (−ΔΔCT) method was used to analyze the relative transcript levels for different experiment groups.

### 2.7. Statistical Analysis

Principal components analysis (PCA), the multivariate analysis tool, is used to reduce a set of original variables and to extract a small number of latent factors (principal components (PCs)) for analyzing relationships among the observed variables. PCA was performed to evaluate variations of gene expression in response to ethephon. Generally, there are three approaches used: Cattell scree test, Kaiser Criterion, and variance explained criteria. We used the scree plot method to assess the number of PCs to be retained. Loading plot, the two-dimensional plane formed by the two first principal components, is the most informative in PCA. The loading plots display the relationships among the detected compounds. The loading of PC1 and PC2 against each other shows the summary of the relationship among variables [30].

All experiments were conducted with three replicates. Statistical analysis was performed using PCA and one-way analysis of variance (ANOVA) followed by SPSS17.0. Differences between treatments were separated by the least significant difference (Duncan) test at a 0.05 probability level. The values are mean ± SD for three samples in each group.

## 3. Results

### 3.1. Effect of Ethephon on Endogenous Ethylene Accumulation and Vinblastine Content

**Figure 2:**
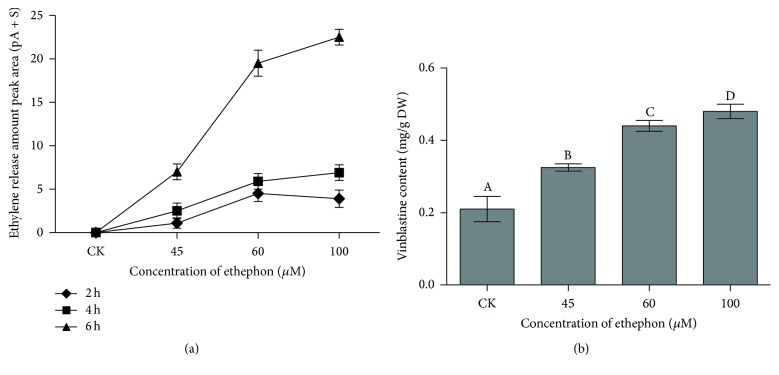
The effect of different concentrations of ethephon on endogenous (a) ethylene release amount and (b) vinblastine contents in* Catharanthus roseus*. The results shown are the means of three replicates; bars represent SE. Different letters indicate significant differences among treatments (*P* < 0.05).

We firstly verified the effect of exogenous application of ethephon, which is absorbed into plants and converted into ethylene, on endogenous ethylene release in* C. roseus* seedling. The results showed that endogenous ethylene release was promoted with the treatment time and concentration of ethephon (*P* < 0.05) (Figure 2(a)). This indicates that the increase of vinblastine accumulation may result from the high concentration of ethephon. Then, the vinblastine contents were measured after* C. roseus* plants were treated by different concentrations of ethephon for 6 h (as is shown in Figure 2(a), we found that this is the best treatment time for* C. roseus* to produce alkaloids). Increasing concentrations of ethephon and up to 100 *μ*M ethephon concentration resulted in enhanced vinblastine content. Treatment with 100 *μ*M ethephon induced vinblastine content about 2 times that in the control group (Figure 2(b)) (*P* < 0.05).

### 3.2. Expression of Genes and Accumulation of Alkaloids in Response to Ethephon

**Figure 3:**
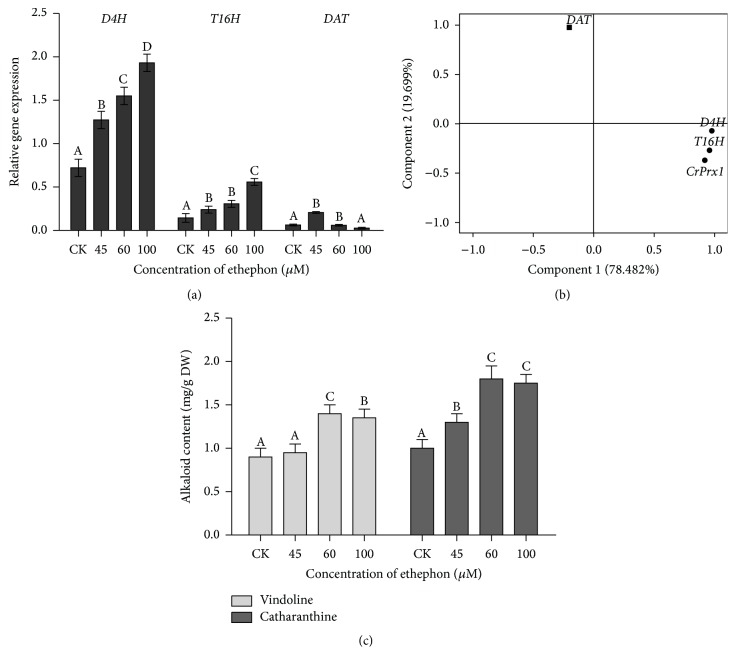
Expression of genes and accumulation of alkaloids in response to ethephon. (a) Relative expression of* DAT, D4H*, and* T16H *genes in* Catharanthus roseus*. (b) Loading plot of PCA. The first principal component (PC1) was the expression of genes* D4H, T16H*, and* CrPrx1*, which were shown as ●. The second principal component (PC2) was the expression of gene* DAT*, which was shown as ■. (c) Vindoline and catharanthine contents in* Catharanthus roseus*. The results shown are the means of three replicates; bars represent SE. Different letters indicate significant differences among treatments (*P* < 0.05).

The contents of vindoline and catharanthine in seedlings of* C. roseus* were also quantified. As shown in Figure 3(a), treatment with 60 *μ*M or 100 *μ*M ethephon increased the production of vindoline and catharanthine of* C. roseus* seedlings about 2-fold, compared with the control. At the transcriptional level, the expression of* D4H* and* T16H* genes followed a similar trend to that of vindoline (Figure 3(a)); expression of* D4H* and* T16H* genes was found to be 3 times more in response to high concentration of ethephon (60 *μ*M and 100 *μ*M), while the expression of* DAT* gene was 3 times more in response to low concentration of ethephon (45 *μ*M) (*P* < 0.05).

As indicated by the loading plot (Figure 3(c)), principal component analysis (PCA) showed two principal components, which can explain 98.181% of the variance of the four downstream TIA pathway genes and serve as high concentrations of ethephon-dependent (*D4H, T16H*, and* CrPrx1*) and low concentrations of ethephon-dependent (*DAT*) variables.

### 3.3. Effect of Ethephon on Redox State 

#### 3.3.1. Effect of Ethephon on H_2_O_2_ Accumulation in* C. roseus* Leaf

**Figure 4:**
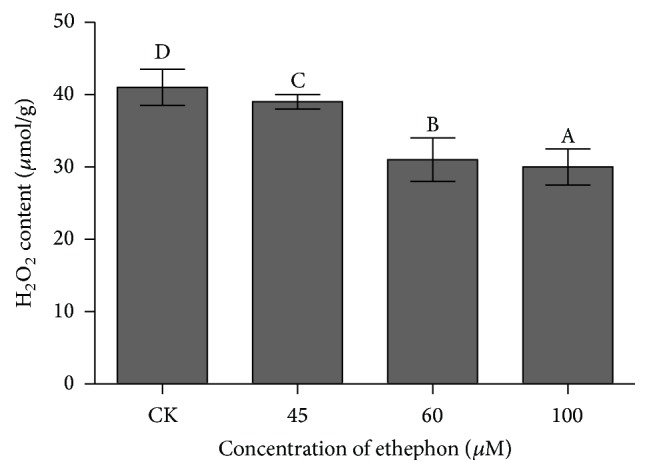
The effects of different concentrations of ethephon on H_2_O_2_ accumulation in* Catharanthus roseus*. The results shown are the means of three replicates; bars represent SE. Different letters indicate significant differences among treatments (*P* < 0.05).

The key function of plant PRXs (POD) is to oxidize phenolic substrates at the expense of ROS, mainly H_2_O_2_. To clearly identify the defense level of POD, the accumulation of H_2_O_2_ in* C. roseus* was also measured (Figure 4). Compared with the control plants, H_2_O_2_ product was inhibited by application of ethephon (*P* < 0.05). We observed that ethephon authentically reduced H_2_O_2_ accumulation in* C. roseus* leaf, and high concentration of ethephon significantly reduced H_2_O_2_ accumulation. With the treatment with 100 *μ*M ethephon, H_2_O_2_ product was 2-fold less in comparison with contrast (*P* < 0.05). This suggested that POD activity decreased H_2_O_2_ accumulation in* C. roseus* leaf.

#### 3.3.2. Effect of Ethephon on Peroxidase Activity

**Figure 5:**
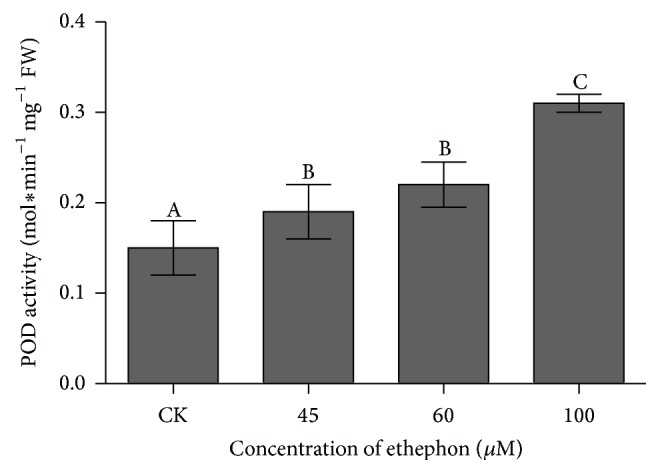
The effects of different concentrations of ethephon on peroxidase activity in leaves of* Catharanthus roseus*. The results shown are the means of three replicates; bars represent SE. Different letters indicate significant differences among treatments (*P* < 0.05).

In* C. roseus*, POD was shown to be responsible for the dimerization reaction between catharanthine and vindoline to produce *α*-3′,4′-anhydrovinblastine, the precursor of the natural antitumoral products, vinblastine and vincristine. To determine the effect of ethephon on peroxidase (POD) of* C. roseus*, different ethephon concentrations were added to the media (Figure 5). It is noticed that POD activity had a lasting upward trend and showed peak of maximal activity in the seedlings treated by 100 *μ*M ethephon (*P* < 0.05).

### 3.4. Effect of Ethephon on* CrPrx1* Transcript

The transcript level of* CrPrx1* in* C. roseus* including control and treated by ethephon at 0 h, 12 h, and 24 h was quantified (Table 2). In comparison to vinblastine content, the exposure of seedlings to ethephon led to a positive effect on the transcription of* CrPrx1* (*P* < 0.05). Responding to these concentrations of ethephon, additive transcription of* CrPrx1* was observed obviously at both 12 h and 24 h and the increment was prominent at 100 *μ*M ethephon. Moreover, the* CrPrx1* transcript level at 24 h was about 2 times higher than at 12 h; particularly, maximal steady-state amounts of* CrPrx1* transcripts were detected in seedlings treated by 100 *μ*M ethephon.

### 3.5. The Loading Plot Combination with Correlation Analysis

**Figure 6:**
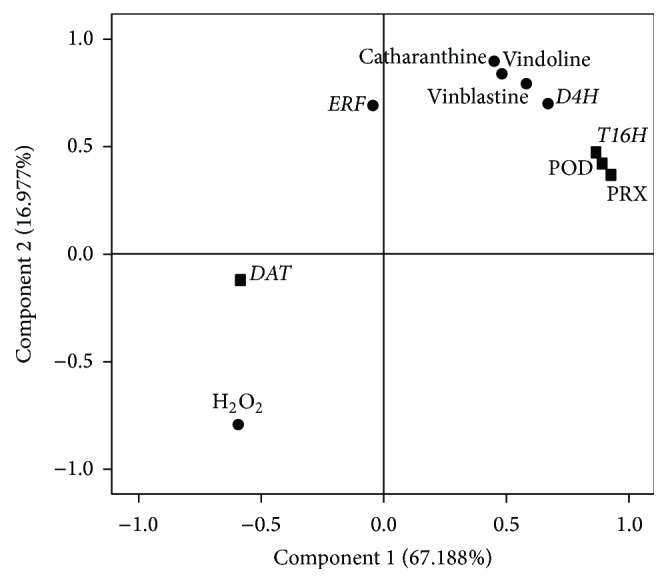
Loading plot of PCA. The first principal component (PC1) was the expression of gene* D4H *and the content of vinblastine, vindoline, and catharanthine and the activity of H_2_O_2_, which were shown as ●. The second principal component (PC2) was the expression of genes* DAT, CrPrx1, T16H,* and* ERF* and the activity of POD, which were shown as ■. The results shown are the means of three replicates; bars represent SE.

We also analyzed the correlation between alkaloids and the transcript levels of genes (the downstream TIA pathway genes and ethylene responsive factor (*ERF*) gene) (Figure 6). As indicated by loading plot (Figure 6), there were two principal components, which can explain 84.164% of the variance of the ten factors and serve as the first principle component (*D4H,* vinblastine, vindoline, catharanthine, and H_2_O_2_) and the second principle component (*DAT, T16H, CrPrx1, ERF*, and POD) variables. H_2_O_2_ presented a significant level of negative correlation in vinblastine accumulation during ethephon treatments, while* ERF* presented a significant level of activity in TIAs accumulation during ethephon treatments. In addition, with the loading plot combination with correlation analysis (Table 2), the results revealed that* CrPrx1* transcript was significantly correlated with vinblastine content and POD activity (*P* < 0.01).

## 4. Discussion

From the present study, it can be concluded that vinblastine biosynthesis influenced by ethephon has a specific relationship with the downstream TIA pathway genes. The result revealed that exogenous ethephon induced a slight increase in endogenous ethylene synthesis (Figure 2(a)). A significant amount of evidence revealed that the regulator had a close relationship with plant growth or secondary metabolism [31]. Tadiello et al. found that all plant tissues are able to produce ethylene, which has been established to modulate a number of important plant physiological activities [32]. The use of elicitors to promote secondary metabolism in plant cell and tissue cultures has become a common practice [33]. Therefore, it is not surprising that ethephon which is converted into ethylene upon metabolism by plants influenced vinblastine accumulation in* C. roseus* (Figure 2(b)). We noticed that vinblastine content was gradually increasing and exceeded the control group at 45~100 *μ*M ethephon. The level of* CrPrx1* transcript is similar as a trend of vinblastine content of* C. roseus* treated by ethephon (Table 2). Furthermore, through analyzing the correlation between vinblastine and transcription of the downstream TIA pathway genes (Figure 6), we found that the results have significant correlation with each other. As the common precursor of all dimeric alkaloids, *α*-3′,4′-anhydrovinblastine (AVLB) was converted into vinblastine through coupling of the monomeric precursors vindoline and catharanthine [7]. Peroxidase was proposed to be a channeling mechanism for the peroxidase mediated-vacuolar synthesis of AVLB [4, 6]. Our data also indirectly indicated that, on the molecular level, vinblastine biosynthesis had a close correlation with peroxidase.

POD is the key enzyme catalyzing vinblastine production. The data revealed that POD activity depending on* CrPrx1* transcript was assuredly promoted by ethephon. From the result of the three alkaloids and POD activity (Figures 2(b), 3, and 5), it was shown that vinblastine content was more sensitive to the activity of peroxidase; therefore, POD activity played a principal role in vinblastine synthesis. Under 60 *μ*M ethephon treatment, vindoline and catharanthine contents were a little higher than that of 100 *μ*M ethephon, but vinblastine content was decreased possibly leading to lower POD activity. We analyzed the correlation between POD activity and transcription of* CrPrx1* (Table 2), and the results also had significant correlation. POD and* CrPrx1* are small part of a complex network of factors subjected to different regulation programs in the TIA pathway and this pathway is not only a single enzyme matching the regulation [3]. Researchers enabled the observation of a clear correlation between the AVLB and* CrPrx1* transcript levels, but details of the process where* CrPrx1* regulated vinblastine biosynthesis are not clear. In addition, POD, dependent on* CrPrx1* transcript, has defense and catalyzing vinblastine biosynthesis mechanism; thus, it plays a more complex role in the process. Our results demonstrate that ethylene regulates vinblastine accumulation via controlling* CrPrx1* transcript and protein which catalyze precursor of vinblastine synthesis at the molecular level. The spatial, temporal, and inducible formation of secondary metabolites and the transcripts of corresponding biosynthetic genes are under tight regulation at different levels, in which transcriptional regulation via transcription factors has been investigated intensively [31]. Guo et al. found that activation of* EIN3* (*ethylene insensitive 3*) increased peroxidase (POD) activity through the direct transcriptional regulation of PODs expression. Accordingly, ethylene pretreatment or* EIN3* activation was able to preclude excess ROS accumulation and increased tolerance to salt stress [34]. In fact, not all regulation is at the level of the EIN3-like transcription factors, and downstream ERF and EDF transcription factors greatly increase the potential points for interaction and cross talk of ethylene signaling with other pathways [23]. Therefore, we tested the expression of ethylene responsive factor (ERF) and investigated the possible regulatory way, by which ethylene controls gene transcription. As shown in Figure 6, the* ERF* mainly participates in the accumulation of TIAs in response to ethephon treatment. This suggests that* ERF* may be the central hub of the POD and* CrPrx1* and, thus, may be actively involved in accumulation of TIAs in response to ethephon.

In vitro, a range of secondary metabolites including phenols, amines, indoles, alkaloids, and sulphonates may act as reductant substrates of class III peroxidases (Prxs), at the expense of H_2_O_2_, which has also emerged as a pivotal molecule in the responses of a range of biotic and abiotic stresses [35]. In this work, we showed increased accumulation of vinblastine, vindoline, and catharanthine with treatment with ethephon. Meanwhile, transcription of the peroxidase (POD) encoding gene* CrPrx1* is also increased by ethephon treatment, accompanied by increased POD activity and decreased H_2_O_2_ content. POD is generally considered to be merely a peroxide-detoxifying enzyme, especially H_2_O_2_ [8]. Hence, H_2_O_2_ accumulation is the evidence to indirectly prove POD and vinblastine synthesis conditions. If this inference is established, H_2_O_2_ accumulation must conform to the trend of POD activity level. According to the result of H_2_O_2_ accumulation, H_2_O_2_ content was reduced by ethephon; therefore, the result consisted with the above reasoning. Some research indicated that H_2_O_2_ content was correlated with vinblastine accumulation [36, 37].

The role of ethylene in plant defense is controversial as it contributes to resistance in some interactions [38, 39] but promotes disease in others [40–42]. Therefore, we suppose that vinblastine may be suppressed by trace of ethephon, while being promoted by higher concentration of ethephon. It should be noted that this study does not provide enough data to understand the behavior of ethylene in these systems [5]. Ethylene interacts with other plant developmental pathways and the mechanisms must be studied in order to clarify these issues in more detail [23].

## Figures and Tables

**Table 1:** Primers used in qRT-PCR for validation of differentially expressed genes.

d4h-F	GACTTGAACTTTCATGCTGCTACAC	25
d4h-R	TCTCATCAAAAGCCTTCAATTCC	23
dat-F	AATCCCTCAGCCGCTATAACC	21
dat-R	ACGGATACGCACGTTTGGTAT	21
CrActin-F	CTATGTTCCCAGGTATTGCAGATAGA	26
CrActin-R	GCTGCTTGGAGCCAAAGC	18
CrT16H-F	GCTTCATCCACCAGTTCCAT	20
CrT16H-R	CCGGACATATCCTTCTTCCA	20
Crprx-F	GCAACATCTCCCAGACCACA	20
Crprx-R	GTTCTCCCAACACTATGAGCACC	23
ERF-F	CACCTCCAATGGCTGCTTTT	20
ERF-R	TCGCTGCCTGCTCTTCTTCT	20

**Table 2:** The relative transcript level of *CrPrx1* and the correlation with vinblastine and POD activityafter 12 or 24 h of treatment with ethephon to *C. roseus*. The results shown are the means of three replicates; bars represent SE.

	CK	+45 *μ*M	+60 *μ*M	+100 *μ*M	Vinblastine	POD activity
12 h	1	2.181 ± 0.021^a^	2.266 ± 0.042^a^	3.249 ± 0.148^b^	0.918^*∗∗*^	0.940^*∗∗*^

24 h	1.765 ± 0.005^a^	2.354 ± 0.019^b^	3.434 ± 0.040^c^	6.714 ± 0.146^d^	0.816^*∗∗*^	0.951^*∗∗*^

Different letters indicate significant differences among treatments (*P* < 0.05). ^*∗∗*^Correlation is significant at the 0.01 level.

## Abbreviations

AVLB: *α*-3′,4′-Anhydrovinblastine
*CrPrx1*: * Catharanthus roseus* peroxidase
*DAT*: Acetyl-CoA: 4-O-deacetylvindoline-4-O-acetyl-transferase
DEPC: Diethylpyrocarbonate
*D4H*: Desacetoxyvindoline-4-hydroxylase
EDTA: Ethylenediaminetetraacetic acid
*ERF*: Ethylene response factor
MMLV: Moloney murine leukemia virus
POD: Peroxidase
RT-PCR: Reverse transcription-polymerase chain reaction
TIAs: Terpenoid indole alkaloids
*T16H*: Tabersonine-16-hydroxylase
PCA: Principal component analysis
PVP: Polyvinyl pyrrolidone.
